# Multiscale Modelling of the Electrocatalysis of Microenzymes: Insights Into the Performance of Microperoxidase at an Aqueous/Graphene Interface and Their Exploitation to Improve Performance

**DOI:** 10.1002/advs.76485

**Published:** 2026-07-20

**Authors:** Milan Mijajlovic, Cheng Hu, Yuhui Sun, Matthew J. Penna, Meisam V. Kiamahalleh, Wenrong Yang, Mark J. Biggs

**Affiliations:** ^1^ School of Chemical Engineering and Advanced Materials Newcastle University Newcastle upon Tyne UK; ^2^ School of Materials Science and Engineering Key Laboratory for Liquid‐Solid Structural Evolution and Processing of Materials (Ministry of Education) Shandong University Ji'nan Shandong P. R. China; ^3^ UniSA STEM University of South Australia Adelaide SA Australia; ^4^ School of Manufacturing Materials and Mechatronics RMIT University Melbourne VIC Australia; ^5^ School of Chemical Engineering The University of Adelaide Adelaide SA Australia; ^6^ School of Life and Environmental Science Centre For Sustainable Bioproducts Deakin University Waurn Ponds VIC Australia; ^7^ School of Engineering and Physical Sciences Heriot‐Watt University Edinburgh UK

**Keywords:** bioelectrocatalysis, Density Functional Theory (DFT), electron transfer, graphene, microenzymes, microperoxidase‐11 (MP‐11), Molecular Dynamics (MD), projector operator diabatization (POD)

## Abstract

There is great interest in systems where enzymes are adsorbed on and act at electrode surfaces, including enzymatic biofuel cells, bioreactors, and biosensors. Although studied extensively since the 1960s, progress is hindered by a lack of molecular‐level understanding of enzyme adsorption and the associated electron transfer processes. Herein, we present a multiscale approach combining molecular dynamics (MD) simulation to elucidate the former and density functional theory (DFT) for the latter. The demonstration focuses on a system involving microperoxidase‐11 (MP‐11), a microenzyme made up of a Fe‐containing heme ring attached to an 11‐residue scaffold, a graphene electrode, and a neutral‐pH saline solution. Adsorbed MP‐11 was found to adopt six possible heme configurations, defined by the iron‐to‐graphene distance and the angle between the heme and graphene normals. The electron transfer rate constants, evaluated by combining MD and DFT results, and their variation with overpotential are comparable to the limited experimental data. The results also illustrate how the new multiscale approach can suggest experimental strategies, from targeted protein engineering to changes in substrate conditions, for improving the electrocatalytic performance of MP‐11/graphene and, more broadly, of other microenzyme/electrode systems of interest across bioelectrocatalysis, biosensing, and bioenergy.

## Graphical abstract

A novel multiscale MD/DFT framework to elucidate microenzyme adsorption and electron transfer is reported. This method reveals distinct configurations for microperoxidase‐11 on graphene and those best suited for electron transfer. Rate constants align with the limited experimental data, establishing this framework as a tool for optimizing microenzyme electrocatalytic technology and a foundation for extension to enzyme systems in future.

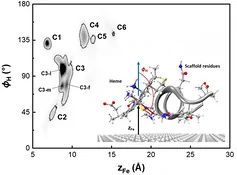

## 1 Introduction

The past six decades has seen extensive research focused on the electrocatalytic activity of enzymes immobilized on electrodes [1, 2] to enable highly sensitive and specific sensors [3, 4], including self‐powered biocompatible devices for in vivo monitoring, biofuel cells capable of generating sustainable electricity from diverse fuels [5, 6], and bioreactors for the production of chemicals with exceptional selectivity [7, 8], even from waste fluid streams. The appeal of these bioelectrocatalytic technologies stems largely from the abundance and ready availability of enzymes and their ability to operate at mild temperature, pressure, and pH compared to synthetic alternatives. Together, these attributes promise cleaner energy, sustainable chemical manufacture, and smart point‐of‐care solutions at lower cost and greater accessibility. That said, several hurdles must be overcome before this potential is realized more routinely. Key challenges include short operational lifetimes due to the natural fragility of enzymes, and low energy and/or product yields arising from limited enzyme loading on the electrode surfaces—constrained by the large size of enzymes relative to their active sites—inefficient electron transfer and other factors (e.g., incomplete fuel oxidation in biofuel cells) [1−358]. Despite decades of effort to overcome these hurdles, progress has been incremental, largely because current technology development strategies rely heavily on empirical, trial‐and‐error approaches [9, 10].

Whilst advances in the field have been very much grounded in the laboratory, molecular modelling techniques such as Monte Carlo (MC) and molecular dynamics (MD) simulation using course‐grained and all‐atom representations with empirical force fields have been increasingly used over the past two decades to improve understanding of enzymes on electrodes [10, 11]. These classical molecular simulation models have been used to predict the position of enzyme active site(s) relative to the electrode, which affects significantly the electron transfer between the enzyme and electrode as well as the accessibility of the active site to the substrate, and the change in enzyme “shape,” which can either enhance or diminish substrate accessibility to the active site. These results can inform the design of improved bioelectrocatalytic technologies, and this will increasingly become the case as the power of computers grows into the future. That said, classical molecular modelling does not provide the complete picture—it says nothing about the electron transfer process either within the enzyme or between it and the electrode. Electron transfer is only revealed through first‐principles molecular modelling methods such as density functional theory (DFT). This has been far less commonly applied in the bioelectrocatalytic context. Petrenko and Stein used DFT to determine the likely electron pathway within an [NiFe]‐hydrogenase adsorbed on graphite and the associated rate [12, 13], and the impact of a point mutation along the likely pathway on the rate [13]. Whilst the determined rates generally matched experiment, the model involved a number of significant simplifications, including adopting cluster models focused only on potential electron transfer pathways determined through Brownian dynamic simulations of a rigid representation of the enzyme adsorbing on the electrode in an implicit solvent. More recently, Futera and co‐workers studied electron transfer through a small tetraheme cytochrome sandwiched between two gold electrodes in the absence of a solvent with a particular focus on the crossover from coherent electron tunneling to an incoherent hopping transport mechanism [14]. Whilst the modelling of the electron transfer process in their work is superior to that of Petrenko and Stein, it was recognized that the four adsorbed states considered may well not be representative of those that would actually exist due to limitations with the MD simulation protocol used to obtain them. A third group has used DFT to study electron transfer between a number of cytochrome c553 variants and graphene [15, 16]. Whilst they did not determine the electron transfer rate, rather interestingly, as in the work reported herein, they found that electron transfer between the cytochrome and graphene is significantly affected by the orientation of the cytochrome's heme ring to the graphene layer, with a “tilted” orientation (i.e., partway between edge‐on and flat‐on) being better.

In this paper, we report for the first time a multiscale coupling of classical MD simulation with first principles (DFT) molecular simulation to link bioelectrocatalytic system performance to the chemical characteristics of the system. This is done in the context of microperoxidase‐11 (MP‐11, Figure 1) [17, 18, 19], which is one of the smallest peroxidase‐active heme‐peptides [20], a microenzyme as defined by Salgado et al. [21] Despite its minimal size, MP‐11 retains the activity of catalyzing oxidation reactions involving peroxides, and has been used in biofuel cells [22, 23, 24], bioresponsive logic circuits [25, 26], and biosensors [27, 28, 29, 30, 31, 32, 33, 34, 35, 36]. Its small size is also what makes the demonstration here tractable: it permits system‐wide MD sampling of adsorption and a first‐principles treatment of the microenzyme and the electrode within reasonable computational resources, opening up use of the approach in the design context, which is the primary motivation for the work reported here. The electrode material adopted here is graphene, which is seen increasingly as the basis for bioelectrocatalytic technologies both involving MP‐11 [37, 38, 39, 40, 41] as well as enzymes more widely [5, 42, 43]. In the remainder of the paper, we first provide a detailed description of the proposed multiscale approach. This is followed by a presentation of the results obtained from applying the multiscale approach to MP‐11 adsorbed at the interface between graphene and a pH‐neutral saline solution. The results include the MP‐11 adsorption mechanism, the dominant adsorbed conformations, and the associated electron transfer performance and comparison with experiment. Drawing on these results, the paper is concluded with a discussion of how the multiscale approach may be used to suggest practical strategies for improving the electrocatalytic performance of the MP‐11/graphene system that in principle could be applied more widely.

**FIGURE 1:**
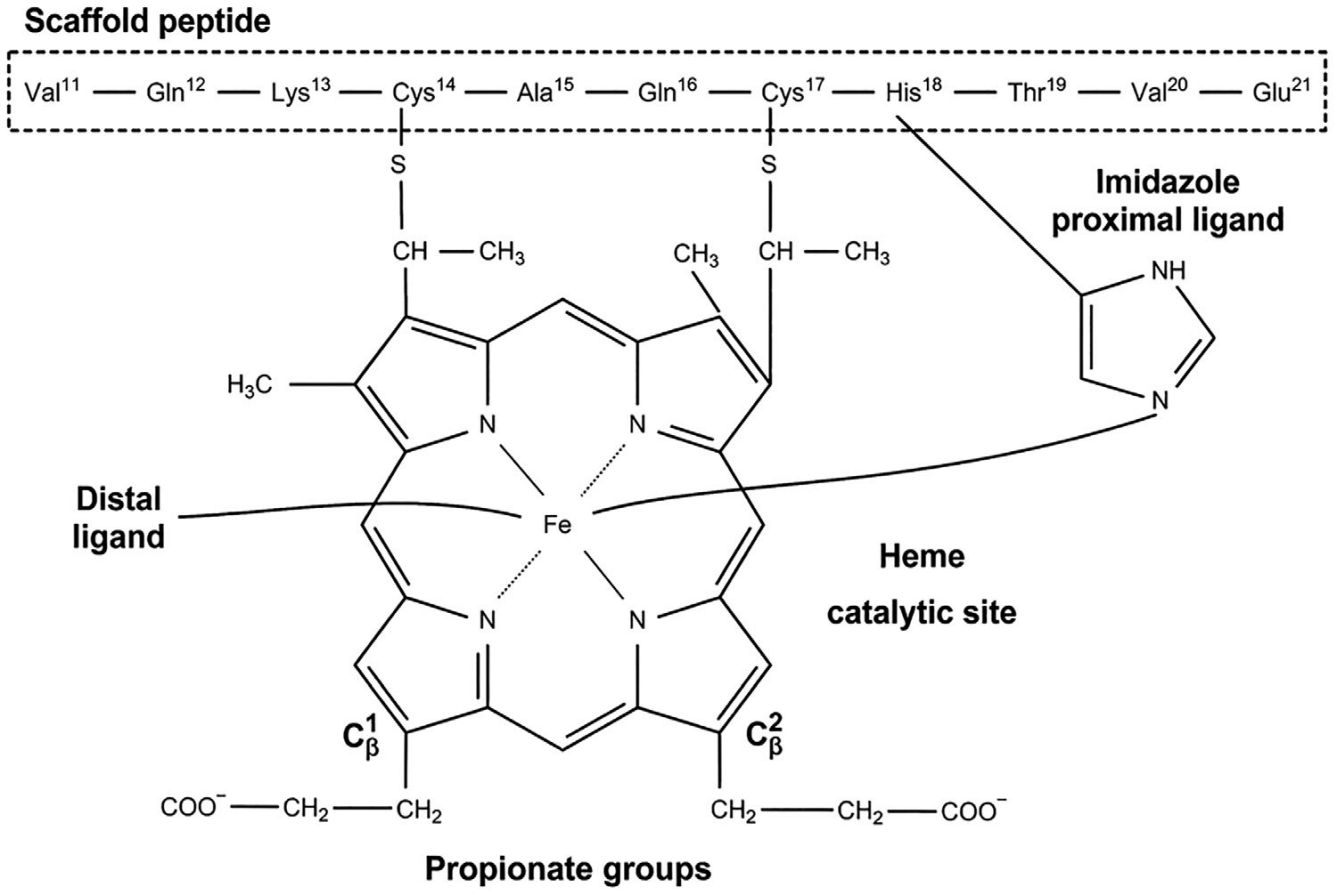
Structural formula of the MP‐11 molecule.

## 2 Methods

### 2.1 System Studied

The system of focus here is the adsorption of MP‐11 from the bulk phase of a pH‐neutral 0.15 M saline solution to the interface between the solution and a rigid pristine graphene sheet and, thereafter, electron transfer between the latter and the microenzyme. As shown in Figure 1, MP‐11 is composed of a catalytic Fe‐containing heme ring that is covalently bound through C atoms at its edges to the sulfur atoms of the Cys^14^ and Cys^17^ residues in an 11‐residue scaffold peptide. The protonation states of the various functional groups of the MP‐11 molecule were defined by the neutral pH. In addition to the four bonds between the heme's iron atom and its inner nitrogen atoms, the former is also axially coordinated below the heme plane with the N^ε^ atom in the imidazole group of the His^18^ residue (proximal ligand). For the distal ligand above the heme plane, an NH_3_^+^ group was utilized as a computational proxy. Because the forcefield of the classical MD simulations is primarily parameterized for the neutral Fe(II)‐porphyrin complex, this proxy was required to better represent the macroscopic electrostatics and bulk diffusion of the net‐positive Fe(III) resting state. Crucially, during the subsequent first‐principles DFT calculations, the system undergoes electronic relaxation during the self‐consistent field (SCF) procedure, correctly localizing the formal positive charge onto the iron atom as expected. Because the catalytic activity of microperoxidases is grounded in the substitution of the distal ligand with a hydrogen peroxide molecule and subsequent cleavage of the O─O bond in the latter [20, 44, 45], this NH_3_^+^ ligand acts strictly as a resting‐state proxy and does not perturb the pre‐catalytic adsorption and electron transfer properties under investigation.

### 2.2 Overview of Multiscale Modelling Framework

The multiscale framework adopted in the study reported here is composed of two levels connected in series. The first level involves classical molecular dynamics (MD) simulation of the adsorption of MP‐11 from a bulk liquid phase (the saline solution here) to the interface between this phase and a solid surface (the graphene sheet here), as per our prior work [46, 47]. This approach yields system configurations—in the form of clusters of similar peptide conformations—that are then fed into the second level of the framework, which involves use of first principles (DFT) simulations to derive the electronic and electron transfer character of the clusters and the system overall. Greater detail for the two levels of modelling is summarized in the following sections.

### 2.3 MD Simulations of MP‐11 Adsorption

The MP‐11 molecule was modelled at the atomistic level with all bonds being flexible as per the CHARMM27 potential model [48] with the exception of the bonds involving hydrogen, which were treated as rigid using the SHAKE algorithm [49].

The graphene sheet was also modelled at the atomistic level. It was rigid and of infinite extent by virtue of applying in‐plane periodic boundary conditions to a graphene sheet of 62.72 × 53.96 Å^2^ in size. The 1352 carbon atoms in the graphene sheet interacted with the rest of the system through a Lennard‐Jones (LJ) potential with parameters equal to those of aromatic carbon atoms of the CHARMM27 potential model [48].

The saline solution was modelled by around 7200 TIP3P water molecules [50] and a number of Na^+^ and Cl^−^ ions corresponding to a sodium chloride concentration of 0.15 M, with an excess sodium ion also being added to ensure the system as a whole was charge‐neutral.

A total of 57 independent simulations were performed with the initial MP‐11 conformation of each simulation randomly selected from a set of uncorrelated conformations collected during a separate bulk phase simulation of the peptide. For each of the adsorption simulations, the selected peptide conformation was initially placed at a randomly selected orientation with its centre of mass at around 30 Å from the graphene sheet in a periodic cell of equilibrated saline solution initially of 60 Å size normal to the graphene sheet. Water molecules that overlapped the peptide were removed from the system, and any unfavorable steric interactions between the peptide and the remaining solution molecules were eliminated by minimizing the system energy through application of a conjugate gradient method for between 3000 and 5000 steps.

All MD simulations were performed using NAMD [51] in the *NpT*‐ensemble. Only the dimension normal to the graphene sheet was varied in order to maintain the target pressure of 1 atm; this was done using the Langevin piston Nose‐Hoover method [52, 53] with a piston oscillation period of 100 fs and a damping time scale of 50 fs. The temperature was maintained at 300 K through the application of a Langevin thermostat [54] with a damping coefficient of 1 ps^−1^. The equations of motion for the system were integrated using the SHAKE algorithm [49] with a time step of 2 fs. Direct calculation of all non‐bonded interactions was performed up to a cutoff radius of 12 Å with a switching function being applied to smooth interactions between 10 and 12 Å. Particle‐mesh Ewald summation [55] with a mesh spacing of 1 Å was used to calculate long‐range electrostatic interactions.

Each MD simulation was run between 24 and 140 ns, depending on the time required for the MP‐11 peptide to substantially engage with the graphene sheet. The cumulative time of the 57 simulations was over 4 µs. Snapshots of the coordinates of all atoms were saved every 1000 steps in each simulation for subsequent analysis. Hydrogen bond analysis of the saved snapshots was undertaken using the relevant VMD plugin [56] on polar atoms of N, O, and S with a donor‐acceptor distance threshold of 3 Å and an angle deviation cutoff of 20° from 180°.

### 2.4 DFT Simulations of MD‐Identified Configurational Clusters

As discussed in the Results section, using the density of configurational states in a two‐dimensional space defined by the distance of the heme's iron atom normal to the graphene plane (*z_Fe_*,) and the heme's angle to the graphene plane (*ϕ_H_*), clusters of similarly adsorbed MP‐11 states were identified. These two variables were used because of their saliency in electron transfer between heme‐based microenzymes and surfaces. For each identified cluster, a single representative structure was chosen for DFT calculation through a root mean square deviation (RMSD) analysis. This involved extracting from all 57 independent MD simulations the frames that belong to a cluster and assembling them to form a “virtual trajectory.” An RMSD analysis was then performed on this trajectory using each frame as a reference against all others. The frame that yielded the minimum average RMSD was selected as the most representative structure for that cluster. Whilst each cluster was primarily considered through this representative structure, intra‐cluster variation of the electronic behavior was also evaluated for the most ‘distributed’ cluster by performing DFT calculations on structures taken from the extremes of its extent (see Section 3.4 for details).

All DFT calculations were performed at the PBE+DFT‐D3(BJ) level of theory with a DZVP‐MOLOPT‐GTH basis set for the entire system [57, 58, 59], including the entire MP‐11 molecule, the graphene, and the solution. The calculations were performed using the Martyna‐Tuckerman Poisson solver [60]. The ground state of the Fe(III) MP‐11 was found to possess a doublet spin state due to the strong‐field nature of the axial imidazole and ammonia ligands. To ensure computational convergence, an orbital transformation method was used for the SCF calculations within the CP2K software package [61, 62]. The graphene sheet consisted of 512 carbon atoms with a size of 39.35 × 34.08 Å^2^. Periodic boundary conditions were applied in the plane of the graphene sheet. The dimension of the simulation box in the Z‐direction was set to twice the span of the specific MP‐11 structure plus 10 Å.

Explicit solvation of the whole‐charge groups of the microenzyme (4 of, see Figure 1) was included to accurately model the system's electrostatics (various alternatives were modelled to identify the most appropriate representation of the solution within the confines of the computational resource available). Four water molecules were placed within 3.5 Å of each whole‐charge group on the MP‐11 molecule, followed by a structural relaxation. To maintain charge neutrality of the system, a sodium cation (Na^+^) solvated by five water molecules was randomly inserted as a counterion. The positioning of the cation was checked to confirm that it had no effect on the results.

### 2.5 Calculation of the Electron Transfer Rate Constant

The rate constant for heterogeneous electron transfer (*k*_*red*/*ox*_) between the MP‐11 and the graphene electrode was calculated using the Marcus‐Hush theory [63, 64], integrating over the energy states (*ϵ*) of the graphene substrate

(1) $$k_{red/ox}=\frac{2\pi }{\hbar }\int H_{ab}^{2}\left(\varepsilon \right)f_{\pm }\left(\varepsilon \right)\rho \left(\varepsilon \right)F\left(\varepsilon \right)d\varepsilon .$$

The electronic coupling element, *H_ab_* (ε), represents the strength of the electronic interaction between the acceptor state on the MP‐11 (its lowest unoccupied molecular orbital, LUMO) and the donor states of the graphene at a given energy *ϵ*. The term *f*_±_(ε)ρ(ε) describes the availability of these donor states, where *ρ*(*ϵ*) is the density of states for graphene, and $f_{\pm }\;\left(\varepsilon \right)={\left[1+\exp(\frac{\pm \varepsilon }{k_{B}T})\right]}^{-1}\;$ is the Fermi‐Dirac distribution. The positive and negative signs in the exponent correspond to reduction (red) and oxidation (ox) processes, respectively. The final term in this equation, *F*(ε), is the Franck‐Condon factor, which describes the probability of finding the MP‐11 molecule in a configuration suitable for electron transfer. It is represented by a normalized Gaussian probability distribution centered at ε  = *e*_0_ η ± λ with a standard deviation of $\sigma ={\left(2\lambda k_{B}T\right)}^{\frac{1}{2}}\;$. For the reduction of MP‐11, this factor is given by

(2) $$F\;\left(\varepsilon \right)={\left(4\pi \lambda k_{B}T\right)}^{-\frac{1}{2}}\;\exp\left(-\frac{{\left(\lambda -\varepsilon +e_{0}\eta \right)}^{2}}{4\lambda k_{B}T}\right)$$

where *λ* is the reorganization energy and *η* is the applied overpotential. Although the former can be evaluated using molecular simulation, a value of 0.3 eV has been assumed here as it led to calculated electron transfer rates that were comparable in scale to the experimental values of Mazzei et al. [65]. The total probability of finding the MP‐11 state across all energies is unity (i.e. $\int_{-\infty }^{\infty }F(\varepsilon )d\varepsilon =1$).

### 2.6 Projector Operator Diabatization for the Calculation of *H_ab_*

The projector operator diabatization (POD) method [66], also known as block diagonalization, was employed to calculate the electronic coupling element, *H_ab_*. This technique is implemented as a post‐processing module in the CP2K software [67]. The system was partitioned into donor (graphene) and acceptor (MP‐11) “fragments” in POD parlance. The Hamiltonian matrix was then transformed by diagonalizing the blocks corresponding to the donor and acceptor fragments separately. This process yields a representation of the system in terms of localized, diabatic states. The off‐diagonal elements of this transformed Hamiltonian, which describe the interaction between the localized donor and acceptor orbitals, are then taken as the electronic coupling elements, *H_ab_*. The donor state in the systems studied here is the graphene HOMO, while the acceptor state is MP‐11 LUMO, which is a singly‐occupied *d*_π_ orbital that is primarily composed of the heme ring's π orbital mixed with either the *d_xz_* or *d_yz_* orbital of the iron atom.

POD was chosen for its balance of cost and accuracy at the present system size. It only requires a single self‐consistent calculation followed by one block‐diagonalization, avoiding the iterative optimization of constrained DFT (CDFT) or the excited‐state calculations of generalized Mulliken–Hush. POD has also been benchmarked to show that its accuracy is comparable to CDFT and has been applied successfully to closely related systems, including electron transfer in multi‐heme cytochromes and charge transfer at molecule/metal interfaces [67, 68, 69]. Like all DFT‐based diabatization schemes, the PBE functional used here is expected to underestimate the absolute *H_ab_* to a degree typical of GGAs [70]. The quantities underpinning our conclusions, however, are the relative couplings between configurations, for which the distance‐decay (*β* factor) is reproduced far more reliably [67]. The localization of MP‐11 LUMO during the POD analysis is highly sensitive to the basis set and the electrostatic environment. It was found that, with the DZVP‐MOLOPT‐GTH basis used here, explicit solvation of the full charges of the MP‐11 and counterions was essential to prevent incorrect orbital localization and ensure a physically meaningful calculation of the electronic coupling.

## 3 Results and Analysis

### 3.1 Water Structuring at the Water/Graphene Interface

Previous molecular simulation‐based studies of aqueous systems such as that studied here have revealed that water structures at the liquid/solid interface play a significant role in the adsorption process from the bulk liquid phase. This structuring is most marked in strongly interacting systems such as metals [46] and minerals [71, 72], but has also been observed for more weakly interacting surfaces such as graphite [47]. Although the interaction of a single graphene layer with an adsorbate is even weaker than for graphite, density and charge analysis reveals that structuring of the water at the graphene surface is still induced. In particular, as seen from Figure 2a, two water layers with densities greater than the bulk phase are easily discernible, whilst there is even evidence of a very weak third layer. Interestingly, the water densities in these layers are greater than those seen for graphite (see Figure 6 in Ref. [47]) despite the weaker interaction arising from graphene. This is due to the water molecules on either side of the graphene layer interacting through the material; the water layers on either side of the graphene reinforce each other, a phenomenon one of the authors reported on two decades ago in a very different context [73].

**FIGURE 2:**
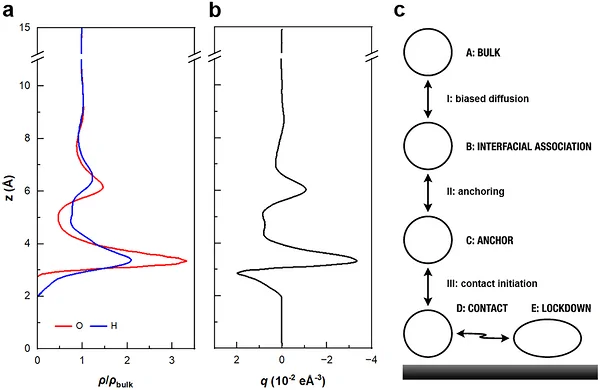
Interfacial water structuring and the stepwise MP‐11 adsorption from the bulk solution: (a) the relative density profiles of oxygen (red) and hydrogen (blue) atoms of water molecules at the interface; (b) the density of electrostatic charge arising from the differing oxygen and hydrogen density profiles; and (c) simplified graphical representation of the adsorption of peptides at liquid/solid interfaces in Ref. [47], where the states along the adsorption pathway are shown with Roman capital letters and the processes that connect them with Roman numerals (see our earlier study^47^ for a more detailed representation and discussion of the adsorption process).

Figure 2a also reveals that there is a difference between the oxygen and hydrogen atom density profiles in the two water layers. This difference reflects an orientational structuring arising from the hydrogen bonds that form between the water molecules. As Figure 2b shows, this difference between the oxygen and hydrogen atom density profiles gives rise to layering of electrostatic charge at the water/graphene interface. The electrostatic interactions between these charged layers and the charged groups on MP‐11 help drive the adsorption of the latter from the bulk solution.

### 3.2 MP‐11 Adsorption Mechanism

A graphical representation of a generalized mechanism of peptide adsorption driven initially by water structuring is shown in Figure 2c [46, 47]. Although initially the peptide is sufficiently far from the graphene that it does not directly interact with it via the truncated LJ interaction associated with its carbon atoms (see Section 2.3 for details of truncation), the water layering and consequent charge separation induced by the graphene that we highlight above lead to the peptide experiencing a force that promotes diffusion towards the graphene (I in Figure 2c). This *biased diffusion* is followed by *anchoring*, in which a peptide group penetrates and remains bound to the second water layer for a reasonable period (II in Figure 2c). Such anchoring may be irreversible in more strongly interacting systems [46], whilst it is not for more weakly interacting systems such as that considered here [47]. In the case where anchoring is sufficiently long‐term, the adsorption process continues by first one group of the peptide coming into direct contact with the solid surface (III in Figure 2c), termed *contact initiation*, with others (but not necessarily all) similarly following in what is termed *lockdown* (IV and onwards in Figure 2c).

The interaction of different groups of the MP‐11 with the water structure and its correlation with the adsorption behavior were further examined through statistical and other analyses of the MD simulation trajectories. Figure 3a shows the propensity for the different groups of the MP‐11 (residues and heme ring) to act as the anchor and contact initiator in the adsorption process shown in Figure 2c. This was generated by counting the number of times during the 57 simulations that each of the groups acted as either an anchor or a contact initiator [46, 47]. A group was counted as an anchor or contact initiator if it was the initial *stable* contact with the second or first water layers, respectively. A group was deemed to be stably connected to a water layer if three or more of its atoms remained for 10 ps or more within a distance from the graphene corresponding to the outer edge of the water layer; this distance was set here at 7.7 Å and 5.0 Å for the second and first water layers, respectively. A total of 265 anchoring and 109 contact initiation events were seen across the 57 simulations. These low per‐trajectory averages (≈ 4.6 and ≈ 1.9 for anchoring and contact initiation, respectively) indicate that once a group anchors, it is highly likely for the peptide to lockdown onto the graphene and remain stably adsorbed, allowing a focus on these adsorbed structures.

**FIGURE 3:**
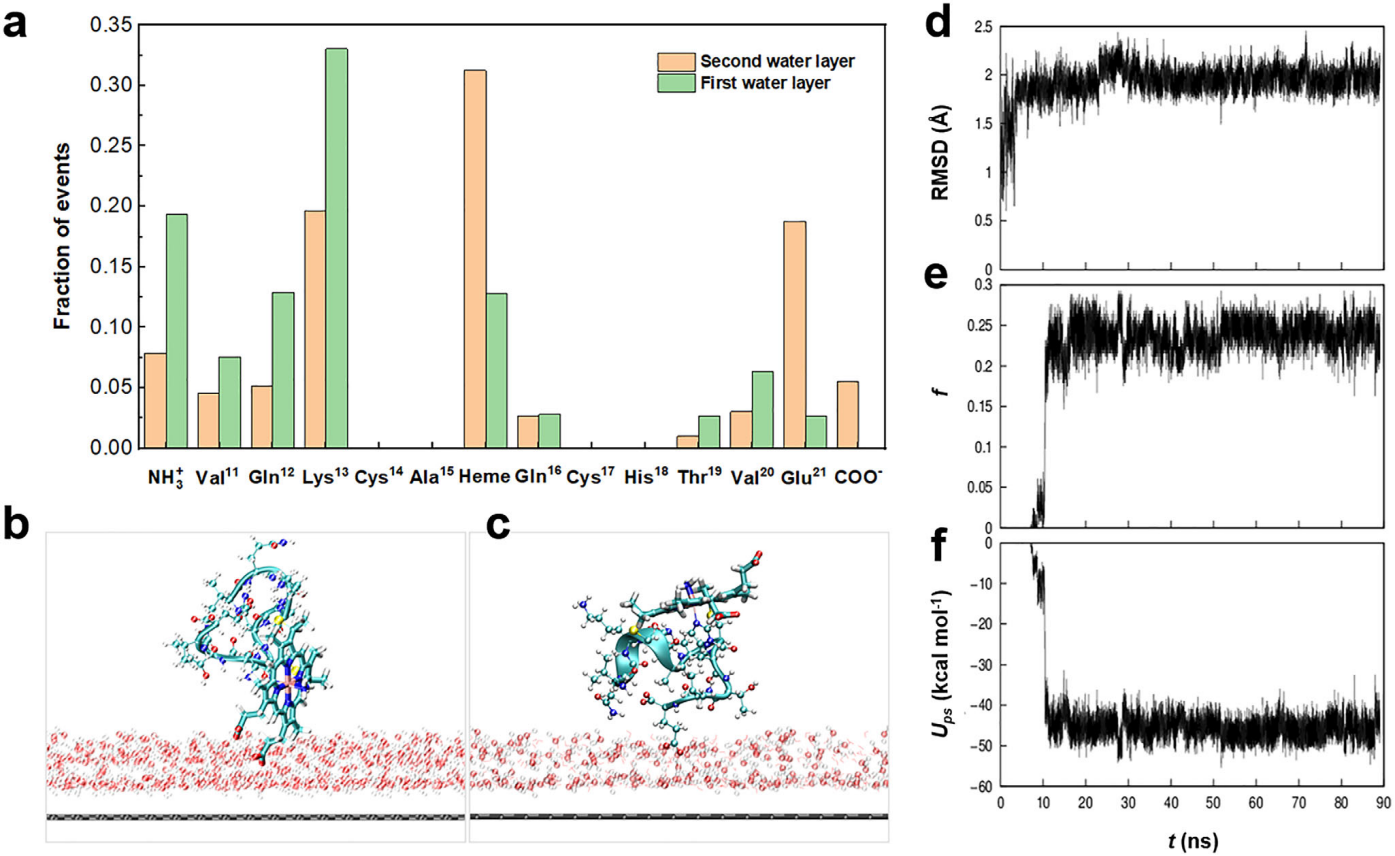
The mechanism of MP‐11 adsorption at the water/graphene interface: (a) the propensity of MP‐11 groups to enter and remain stably within the second water layer (i.e., anchoring) and first water layer (i.e., contact initiation) given as the number of times the group has been observed to be the first one to establish a stable presence in the layer relative to the total number of such events; exemplar simulation snapshots in which the MP‐11 molecule associates with the second water layer via (b) the propionate groups of the heme, and (c) with the carboxylic groups of the Glu21 side chain; the conformational change of MP‐11 from an exemplar adsorption trajectory expressed through (d) the root mean square deviation (RMSD) of its backbone C^α^‐atoms using the initial conformation as a reference, along with (e) the corresponding fraction of peptide atoms in direct contact with the surface, and (f) the potential energy of peptide‐surface interaction.

It is evident from Figure 3a that the heme group has the highest propensity (around 31.3%) to act as an anchor. More detailed analysis reveals that the two negatively charged propionate groups located at the edge of the heme ring account for 78.7% of this (i.e., 24.6% of all anchoring events); an exemplar of one of these anchoring conformations is shown in Figure 3b. This is likely to be due to favorable interaction between these negatively charged groups and the positively charged layers that bracket the second water layer, which are located within the ranges of 3.9 ≤ *z* ≤ 5.6 Å and 6.6 ≤ *z* ≤ 8.2 Å (see Figure 2). The remainder of the heme's anchoring propensity (21.3%) arises from the NH_3_^+^ distal ligand. Once again, this is likely to be due to the attraction of this group to the negatively changed region associated with the second water layer (i.e., 5.6 ≤ *z* ≤ 6.6 Å).

The anchoring propensities of Lys^13^ (19.6%) and Glu^21^ (18.9%) are also significant. The latter residue has a negatively charged carboxylic group in its side chain as well as the zwitterionic C‐term of the backbone, both of which will be attracted to the positively‐charged layers mentioned above; a snapshot showing an exemplar of an anchoring event involving this side chain is shown in Figure 3c. The positive end of the lysine sidechain means the story for Lys^13^ differs in that it will be attracted to the negatively charged layer that sits at the heart of the second water layer (i.e. 5.6 ≤ *z* ≤ 6.6 Å).

The remaining residues play a far more minor‐to‐no role as an anchor. The sidechains of Cys^14^, Cys^17^, and His^18^ being the tether points for the heme ring explains why they never act as anchors. The long sidechain of glutamine combined with its capacity to form hydrogen bonds makes glutamine a potential anchor. This is seen for both Gln^12^ (5.3%) and Gln^16^ (2.7%), although the lower propensity for the latter is presumably due to ‘shielding’ from the heme ring. The very short and hydrophobic nature of alanine's methyl sidechain explains why Ala^15^ never acts as an anchor (it is also shielded by the heme ring). Whilst the sidechains of valine and threonine are longer than that of alanine, they are still relatively short as well as being hydrophobic, both of which would explain their low propensities to act as an anchor. The charged and accessible natures of both termini mean they have a higher anchoring propensity than many of the other residues – 8.0% and 5.7% for the N‐term and C‐term, respectively — despite being relatively small in size.

As seen in Figure 3a, the propensity of the different parts of MP‐11 to act as the graphene contact initiator follows a different pattern from that seen for anchoring. The fact that the positively charged sidechain of Lys^13^ is the most likely to act as the contact initiator (33.0%) is not surprising, given that the first water layer located between 3.1 ≤ *z* ≤ 3.9 Å is negatively charged (see Figure 2). This also explains the relatively high propensities for the positively charged N‐term (19.3%) and the heme distal ligand (9.5%) to act as contact initiators. The sidechain of Gln^12^ also has a reasonable propensity for initiating contact with the graphene (13%), presumably because its NH_2_‐ and CO‐groups are readily available to form strong hydrogen bonds with the densely packed water molecules in the first layer. Valine is also more likely to be involved in the process due to its position at the N‐terminal and its zwitterionic NH_3_^+^ group. Both the heme group and Glu^21^ play a far lesser role in the initiation of lockdown. This is likely to be caused by their negatively charged carboxylic groups, which will be repulsed by the first water layer that carries an overall negative charge (Figure 2b). In addition to this charge effect, the heme group is also relatively rigid and bulky, which hampers its penetration into the densely packed first water layer, at least in short enough time scales to make it a dominant contact initiator. Ultimately, however, as is shown below, further rearrangement of the peptide structure in the lockdown phase allows the heme group to enter the first water layer even though it is less dominant in the initial insertion.

Interestingly, despite the similarity in the nature of the surface to that used in our study of graphite‐binding peptides [47], the lockdown phase did not always proceed in the same stepwise manner. For some of the simulations, in particular those where the interaction between the heme ring and graphene dominated, further substantial conformational change was not observed following contact initiation. This is reflected by the root mean square deviation (RMSD) of the C^α^‐atoms of the scaffold peptide for the entire duration of an exemplar trajectory (Figure 3d). By combining with the time evolution of the fraction of peptide atoms that are in direct contact with graphene (Figure 3e), it can be concluded that much of the conformational change is experienced by the peptide before initiation of the lockdown process (c5 ns for the exemplar simulation). Upon contact initiation at about 6 ns, the fraction of MP‐11 atoms in direct contact with graphene remains low until about 10 ns at which point the rest of the peptide is drawn and locked to the surface. Further simulation out to 90 ns does not see a major change in either the peptide conformation or the fraction of its adsorbed atoms (see Video S1). This sustained engagement is further reflected in the C^α^‐to‐graphene distances of the contacting residues, which remain within the inner water layer throughout the post‐lockdown period (Figure S1), leading to a stable peptide‐surface interaction energy (Figure 3f). While this result may seem counterintuitive given our work reported elsewhere for adsorption of peptides of similar size at the water/graphite interface [47], it should be noted that the structure of MP‐11 is substantially different due to the presence of the bulky, rigid heme ring. This is especially significant for adsorbed configurations in which the heme ring is near the surface, which, as will be seen below, dominate the ensemble of adsorbed structures.

### 3.3 The Heme Configuration of Adsorbed MP‐11

The efficiency of electron transfer between MP‐11 and the graphene is profoundly influenced by the conformation the molecule takes relative to the graphene layer, and particularly so the configuration of the heme group. To facilitate understanding of the heme configuration in the simulations, we have defined two collective variables for the heme as shown in Figure 4a: the distance between the Fe atom and the graphene sheet, *z_Fe_*; and the angle between the normal to the graphene sheet and the bond between Fe and N^ε^ atom of the His^18^ residue, *ϕ_H_*. Although it is understood that the efficiency of electron transfer is improved as the distance between the Fe and the surface reduces, there is far less understanding of what the optimal heme ring orientation is. As shown below, *ϕ_H_* also plays a significant role, particularly for the configurations with a lower *z_Fe_* value when the heme ring is adsorbed edge‐on to the graphene surface. In this scenario, *z_Fe_* and *ϕ_H_* are correlated, and the latter presents a significant impact on the wavefunction overlap (and, hence, the electron transfer efficiency) between the graphene states and the MP‐11 LUMO, which is delocalized on the heme ring. In addition, *ϕ_H_* also determines the reactant's accessibility to the catalytic site. Thus, it is anticipated that the optimal heme ring orientation will balance the need to maximize electron transfer rates on the one hand whilst ensuring its accessibility to the substrate in the solution on the other.

**FIGURE 4:**
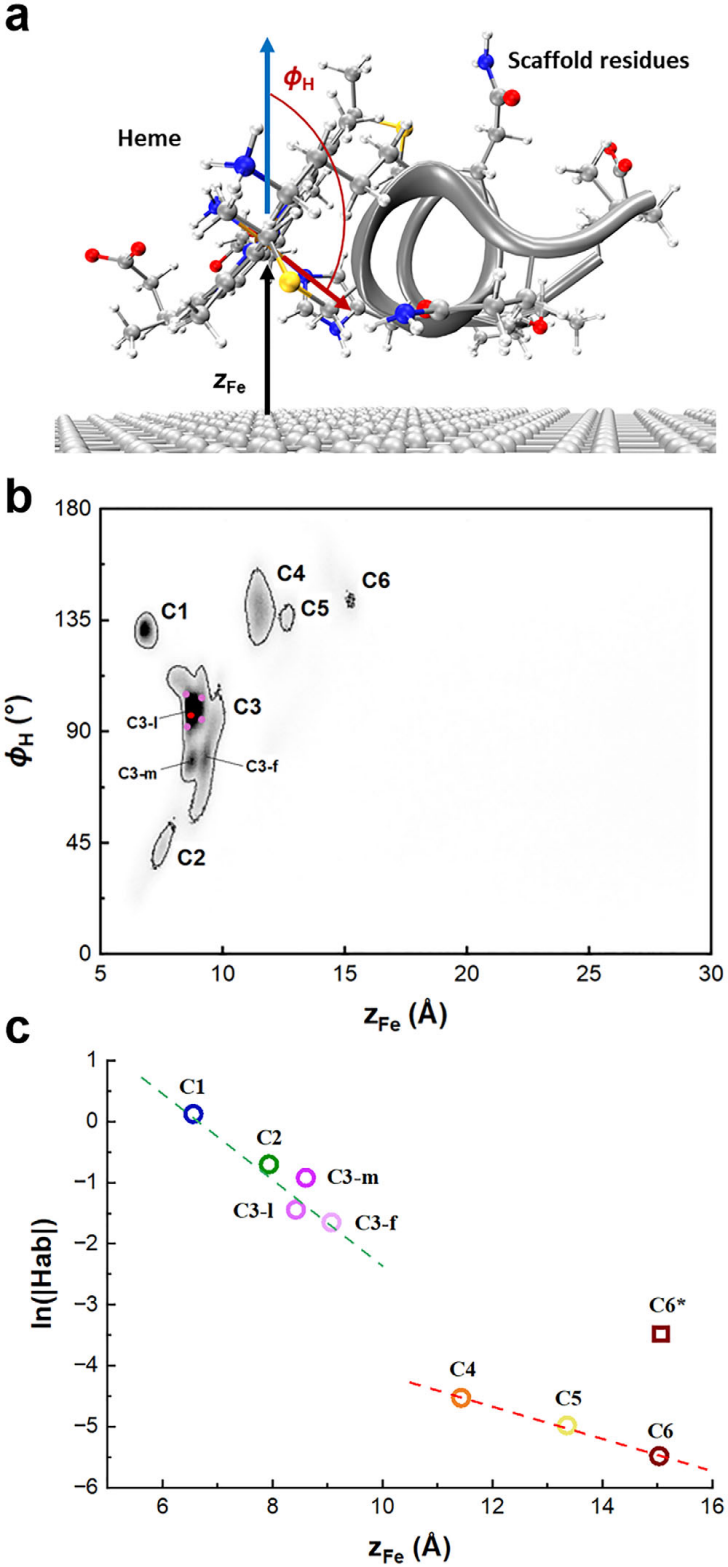
The heme configuration of adsorbed MP‐11 and its impact on interfacial electron transfer: (a) a representative structure that shows the definition of the parameters used to define the heme configuration—distance between the graphene surface and the Fe atom (*z_Fe_*, black arrow), and the *ϕ_H_* angle between the Fe‐N bond vector (Fe to N^ε^ of His^18^, red vector) and surface normal (blue arrow); (b) the probability density of heme configurations in the *z_Fe_‐ϕ_H_* space evaluated using all snapshots from the 57 simulations undertaken, where contours are set at a value of 6.5 ×10^−10^ 1/(Å·°) and the identified clusters of heme configuration and sub‐clusters of the C3 cluster are labeled; (c) relationship between the natural logarithm of the electronic coupling, ln(∣*H_ab_* ∣), and *z_Fe_* for the identified heme configuration clusters C1, C2, C4‐C6 and the three sub‐clusters of the C3 cluster (C3‐l, C3‐m, C3‐f).

As the adsorption of MP‐11 proceeds to the lockdown stage on the graphene surface, the configuration of its heme group becomes significantly constrained. Figure S2 shows the trajectory of the MP‐11 molecule in the *z_Fe_*‐*ϕ_H_* space from an exemplar simulation. This figure shows that the molecule spends some time diffusing in the bulk phase and the orientation of the heme group to the surface varies widely. However, as the molecule moves towards the graphene surface and finally locks down, the orientation of the heme group to the surface becomes increasingly constrained, particularly once the Fe‐to‐graphene distance falls below 12 Å. As the yellow region in Figure S2 indicates, the MP‐11 molecule finally becomes trapped at the interface with the Fe atom limited to a relatively narrow band between 6 and 10 Å from the graphene sheet, restrained by *ϕ_H_* falling between 20° and 80°, respectively.

To generalize this result, Figure S3 presents the recorded snapshots from all 57 simulations in the *z_Fe_*‐*ϕ_H_* space. The diffuse black region away from the graphene confirms that in general the heme group of the molecule in the bulk phase has no preferred configuration relative to the graphene layer. The dense black regions closer to the graphene in this figure also confirm that, more generally, there is a far stronger coupling between the heme ring orientation and distance as MP‐11 gets closer to the graphene layer. This is more clearly seen in the probability density function (PDF) shown in Figure 4b, which reveals that the heme group can take on six distinct configurational clusters when adsorbed at the water/graphene interface, although one of these (C3) may be further subdivided into three sub‐clusters that we term C3‐l, C3‐m and C3‐f. The relative propensities for each of these clusters are shown in Table 1, and their representative structures identified via the RMSD analysis described in Section 2.4 are illustrated in Figure 5.

**TABLE 1:** Calculated |*H_ab_* | and *k* for different heme configurations, and the system overall.

Cluster	*z*_Fe_ (Å)^a^	*Φ* (°)^a^	$\bar{|H_{ab}|}$ (eV)^b^	*k* (s^−1^, λ = 0.3 eV)		Fraction
				*k*_0_	*k*_max_ (×10^4^)^c^	
C1	6.562	132.4	1.130	4420	8510	9.18%
C2	7.936	51.7	0.496	3170	6770	3.67%
C3	‐^d^	‐^d^	‐^d^	1040^e^	3630^e^	67.89%
C3‐l	8.426	98.5	0.237	1150	3900	22.46%
C3‐m	8.607	77.3	0.397	2100	5300	1.42%
C3‐f	9.072	80.1	0.192	162	1100	4.68%
C4	11.431	141.3	0.0108	3.03	7.68	14.93%
C5	13.357	136.2	0.00688	0.137	1.18	3.80%
C6	15.060	143.1	0.00416	0.117	0.451	0.53%
System^f^				1230	3510	

^a^ Values for the conformations taken to represent the clusters (Figure 5).

^b^ Average |*H*_ab_| between MP‐11 LUMO (located on the heme group) and the highest 10 occupied orbitals of graphene.

^c^ *k_max_ is k* at an overpotential of −1 V, inline with experimental practice.

^d^ Representative structures available for three C3 sub‐clusters (Figure 5).

^e^ Propensity‐weighted sum of the three C3 sub‐clusters; the C3 area beyond these sub‐clusters was ignored as it was not possible to clearly define a single or small number of conformations for this region of the heme configurational space.

^f^ Propensity‐weighted sum of all the configurations.

**FIGURE 5:**
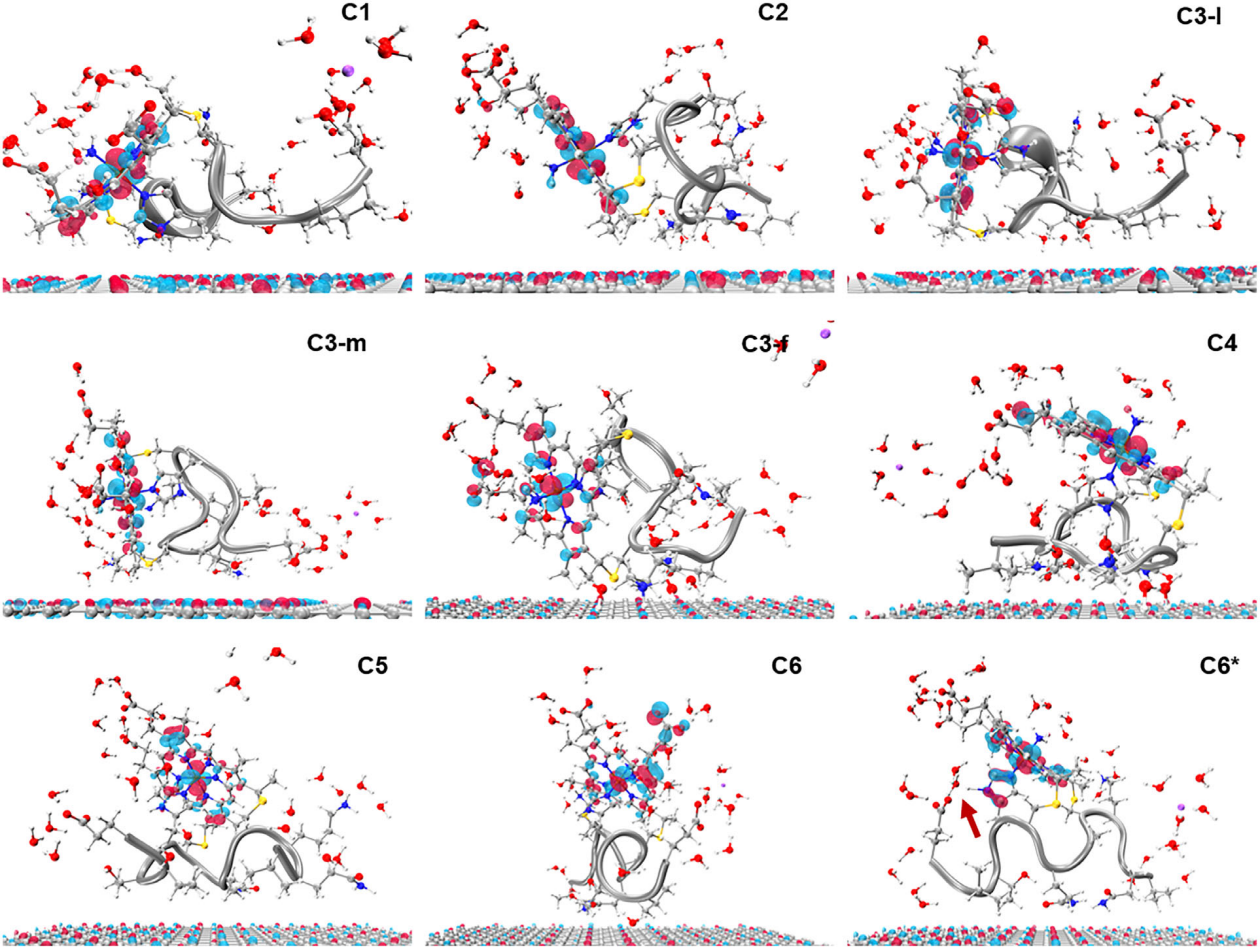
Representative MP‐11 conformations for the heme configurational clusters shown in Figure 4b. The lowest unoccupied molecular orbital (LUMO) of MP‐11 and the highest occupied molecular orbital (HOMO) of graphene, obtained from the POD analysis of electron transfer, are plotted for an isosurface value of 0.03.

The most common cluster is that denoted C3, characterized by the heme ring being more‐or‐less adsorbed edge‐on to the graphene, with its face being accessible to the solution bulk to a greater (higher *ϕ_H_*) or lesser (lower *ϕ_H_*) extent. The C1 and C2 clusters sit closer to the graphene compared to the C3 cluster but share the same heme side‐on configuration as the latter. However, the tilt of the heme ring for C1 and C2 is opposite, resulting in the active site facing the solution bulk (C1) and the graphene (C2), respectively. Whilst the orientation of the heme ring for clusters C4 to C6 is similar to that of cluster C1, they are located significantly further away from the graphene because the scaffold peptide interposes itself between the heme ring and graphene compared to being adjacent for clusters C1 to C3 (see Figure 5).

### 3.4 The Impact of Heme Configuration on Interfacial Electron Transfer

Table 1 includes the average electronic coupling, $\bar{|H_{ab}|}$, evaluated here using POD analysis for clusters C1, C2 and C4‐C6 and the three sub‐clusters of C3, and the standard electron transfer rate constant, *k_0_*, at a temperature of *T* = 300 K using a reorganization energy of *λ* = 0.3 eV [65]. The average electronic coupling is evaluated between the MP‐11 LUMO and the highest 10 occupied orbitals of graphene, as these graphene states are the primary contributors to the electron transfer at low overpotentials (0 to c1 V). This table reveals the average electronic coupling is in the meV range or less for all the clusters. As this is significantly lower than the thermal energy at 300 K (*k_B_T*  = 26 meV), the electron transfer between the graphene and the adsorbed MP‐11 may be considered non‐adiabatic in nature, confirming the applicability of the Marcus‐Hush theory here where the rate constant is proportional to the square of the electronic coupling element (|*H_ab_* |^2^) (*c.f*. Equation 1).

Figure 4c shows that the electronic coupling, and hence rate constant, increase exponentially with respect to the distance between the heme‐bound Fe ion and the graphene surface, as expected for nonadiabatic electron transfer. The C1 and C2 clusters are the most favorable for electron transfer, exhibiting a standard rate constant, *k*_0_, that is more than 5 orders of magnitude greater than that of the least favorable C6 cluster. Although C2 presents an $\bar{|H_{ab}|}$ (and, thus, *k*_0_) value that is comparable to that of the C1 cluster (c44%), the latter may well be more active than their relative rates suggests because its heme configuration makes the Fe ion more accessible to the substrate than is the case for C2 where the heme ring is facing towards the graphene (i.e. the rate of reaction for the C2 configuration is more likely to be mass transfer controlled than the C1 configuration, all else being equal).

It is interesting to note that the rate of change of the electronic coupling (and thus rate constant) with *z_Fe_* for clusters C1‐C3 (indicated by the green broken line in Figure 4c) is greater than that for clusters C4‐C6 (indicated by the red broken line). The heme's π^∗^ antibonding orbital, which makes a substantial contribution to the MP‐11 LUMO (seen from the isosurface plots in Figure 5), is the origin of the higher rate of change for clusters C1‐C3. In particular, at smaller *z_Fe_* associated with these clusters, this orbital increasingly overlaps with the graphene states as the angle *ϕ_H_* deviates further from 90° (i.e., as the plane of the heme ring rotates towards or away from the graphene layer), ‘reinforcing’ the rate of change that arises with varying *z_Fe_*. This reinforcement effect of heme tilt from the edge‐on configuration is also reflected in the electronic coupling of C3‐l, whose *ϕ_H_* is around 90°, siting below the green broken line, whilst that of C3‐m is above this line despite being further away from the graphene because its angle of tilt deviates substantially from 90°.

The large extent of the C3 cluster in the *z_Fe_*‐*ϕ_H_* space suggests the possibility of significant variation in the electron transfer efficiency within the cluster, and Table 1 indicates that this is indeed the case, with the rate constants of the sub‐clusters varying by up to a factor of c10 (compare *k*_0_ of C3‐m and C3‐f in Table 1). Further probing of the variation of coupling (and this rate constant) within the largest of the C3 sub‐clusters (C3‐l), reveals on the other hand, a variation of less than an order of magnitude between the four corners of the sub‐cluster (purple spots marked in Figure 4b, structures shown in Figure S4, and data in Table S1). Returning to the variation between the sub‐clusters, as seen from the structures of C3‐l, C3‐m, and C3‐f (Figure 5), this mainly arises from the tilt of the heme group. One source of this variation has already been highlighted in the previous paragraph. In the case of the C3‐f sub‐cluster, however, its lower electronic coupling (and hence rate constant) arises from the rotation of the heme group pushing the entire moiety further away from the graphene surface.

Initial calculations for the C6 configuration also revealed an “unexpected” behavior, where a greater contribution from the proximal imidazole ligand to the MP‐11 LUMO induced an anomalously high electronic coupling (denoted as C6* in Figure 5). The underlying cause was traced to a deprotonation event that occurred during structural relaxation in the DFT simulation (indicated by the arrow in Figure 5). This occurs when the deprotonated carboxyl group of Glu^21^ is positioned close to the pyrrole‐like nitrogen (N1) of the imidazole ring. The carboxylic acid group attracts a proton from the water chain, initiating a proton relay that ultimately results in the deprotonation of the imidazole nitrogen. The resulting imidazolide anion has an extra electron lone pair, which modifies the frontier molecular orbitals and enhances the contribution of the imidazole ring to the LUMO. This imidazole deprotonation would be unlikely when complete water solvation of MP‐11 is modelled, suggesting that the reduced treatment of the solvation used here, whilst often accurate, may not always be so.

### 3.5 Electron Transfer Rates for the System and Comparison With Experimental Values

Figure 6a shows the variation of the system rate constant as a function of overpotential for three different values of reorganization energy, *λ*, where the system rate constant is evaluated by summing the propensity‐weighted values for the various heme configurations. The shape of these plots is akin to the experimental Tafel plot of reduction reactions obtained by Mazzei et al. [65]. Up to an overpotential of *η* = −1 V for their [MP‐11+H]^2+^ molecule adsorbed on carbon nanotubes (this is where they define *k_max_*, which we will also do here). As simulation allows determination of rates at overpotentials that are higher than those normally accessed experimentally, it can be seen from Figure 6a that the rate continues to increase until it plateaus at an overpotential of around *η* =  – 3 V. This behavior is due to the continuous increase of the graphene density of states, ρ(ε), below the Fermi level [74].

**FIGURE 6:**
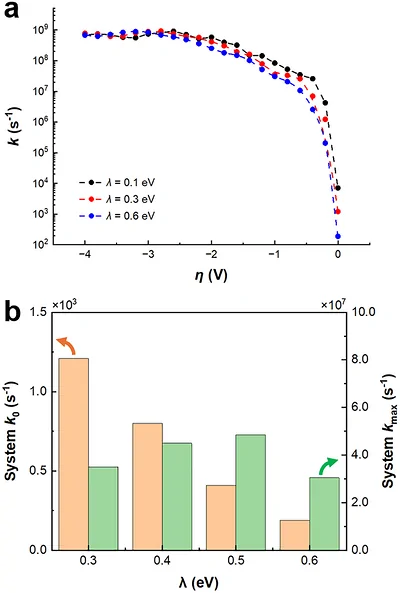
Electron transfer rates for the system: (a) the system rate constant as a function of overpotential at different values of reorganization energy, *λ*; and (b) the impact of *λ* on the system equilibrium rate constant, *k*_0_ (orange, left axis), and ‘maximum’ rate constant, *k*_max_ (green, right axis), taken at an overpotential of −1 V, in line with experimental practice in the field. The dashed lines in (a) were plotted as Bezier curves to serve as visual guides to the overall trends of the data points.

Figure 6a also shows, as per Equation (2), that the rate constant is influenced by the reorganization energy, *λ*, with it tending to decrease as *λ* increases. Figure 6b shows that the standard rate constant is more sensitive to the reorganization energy, varying by around one order of magnitude as *λ* moves from 0.3 to 0.6. This highlights the need for accurate, configuration‐specific determination of reorganization energy in future studies. Figure 6b also reveals rather interestingly that the rate constant determined at an overpotential of *η* = −1 V, *k_max_*, varies non‐monotonically with the reorganization energy. This arises from the discrete, charge‐localized graphene states required by the POD method and would be suppressed if the propensity‐weighted sum of data from individual structures within a cluster were to be used (see the difference between Figure 6a and an exemplar plot from the C1 structure shown in Figure S5, which illustrates even more marked oscillatory behaviour arising from use of a single structure within a cluster).

As shown in Table 2, taking *λ* as 0.3 eV, both the *k*_0_ and *k*_max_ evaluated here are order‐of‐magnitude comparable to the experimental values obtained also using the Marcus‐Hush theory [65, 75]. Here, *k*_max_ is taken as the rate constant obtained at an overpotential of *η* = −1 V, in line with the experimental practice in the field. The deviation from experimental values can be explained by the differences in the systems, such as the net charge of the MP‐11 (influenced by substrate chemistry and pH, see Table 2 for the variation in experimental values caused by MP‐11 charge state), specific electrode material used (e.g., multiwalled carbon nanotubes vs. graphene), and the surface coverage. Indeed, this can be seen more clearly in the even larger differences in the experimental data gathered by Yarman et al. [76] for MP‐11 for different substrates and electrode materials.

**TABLE 2:** Comparison of rate constants determined here with experiment [65, 75].

System	*k*_0_ (s^−1^)	*k*_max_ (×10^7^ s^−1^)	λ (eV)^*^
[MP‐11+H]^2+^ on MWCNT (MP‐11a)	2029 ± 47	11 ± 1.1	0.58 ± 0.09
[MP‐11+H]^3+^ on MWCNT (MP‐11b)	1985 ± 39	1 ± 0.9	0.30 ± 0.05
[MP‐11+H]^2+^ on gold	1080 ± 35	3.0 ± 0.4	1.0 ± 0.1
[MP‐11+H]^3+^ on gold	810 ± 29	0.08 ± 0.01	0.65 ± 0.08
This study—[MP‐11–2H]– on graphene	1230	3.51	0.30

* The experimental values were obtained through fitting the Marcus‐Hush equation to the experimental data, whilst the value for this study is assumed.

### 3.6 Towards Optimizing Electron Transfer

The practice of using molecular modelling to optimize bioelectrocatalytic technologies is in its infancy and has essentially been limited to what classical molecular modelling says about the orientation of the active site to the electrode surface [10, 11]. Whilst the main purpose of this paper is not to do the same for the MP‐11/graphene system—that is for the future—we illustrate here an enhancement of this past practice using the new multiscale approach reported in the paper. The illustration is focused on how the propensity of the best‐performing configuration, C1, could be potentially increased from its “native” c9% (Table 1) at the expense of the vastly more common but far worse‐performing C3 configuration. The multiscale modelling here suggests that if this were to be achieved in its entirety, the equilibrium rate constant would increase by nearly 3‐fold, all else being equal, whilst *k_max_* would rise by more than two‐fold.

To help further focus the discussion, consideration will be restricted to the C3‐l configuration, which is “closest” in the heme configurational space to the C1 cluster, and also accounts for the vast majority of the well‐defined space of the C3 cluster. Analysis of the MD results reveals three main differences between the C1 and C3‐l configurations beyond *z_Fe_* and *ϕ_H_*, namely:

1. The heme rings of the two configurations are “flipped” 180°. This is reflected in Figure 7a, which shows that the $C_{\beta }^{1}$ propionate sits closest to the graphene for the C1 configuration whilst $C_{\beta }^{2}$ is closest for the C3‐l configuration. This difference arises from the conformations in C1 being bound to the graphene through the N‐term (N‐term down), whilst those in C3‐l sub‐cluster are bound by the C‐term (C‐term down).
2. As shown in Figure 7b, the ψ angle of the His^18^ residue is the only dihedral angle in the backbone of the scaffold peptide to substantially differ between the two configurations. This residue is, of course, connected to the heme ring via the imidazole group in the sidechain (Figure 1), and is the first part of a two‐part story around the crucial difference in heme orientation relative to the graphene sheet.
3. The second part of the story is revealed in Figure 7c, which shows the high prevalence in intra‐scaffold hydrogen bonding in the C1 configuration compared to C3‐l. This makes the scaffold sufficiently rigid to support the translation of the change in the His^18^ ψ angle to the heme group (via the imidazole group in its sidechain), causing its orientation to change. Further analysis of the MD data shows that these hydrogen bonds are formed as the MP‐11 molecule approaches the surface rather than when it is adsorbed (Figure S7).

**FIGURE 7:**
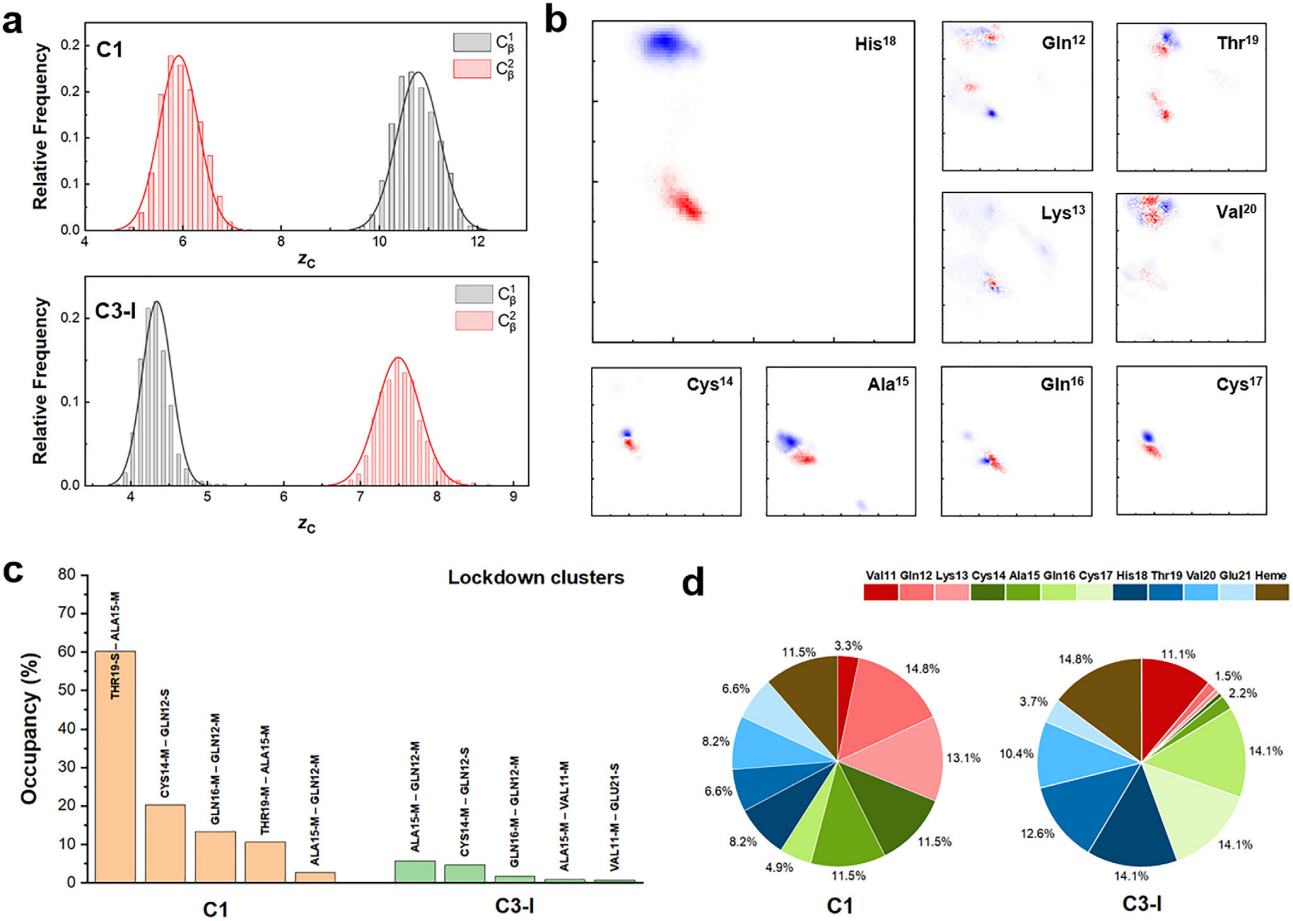
Main differences between the C1 and C3‐l configurations beyond *z_Fe_* and *ϕ_H_*: (a) normal distance of the propionate groups from the graphene sheet for the two configurations; (b) difference in Ramachandran plots^74^ of the backbone dihedral angles (*ϕ*, *ψ*) of the two configurations for the nine inner residues of MP‐11 in the bulk liquid phase and the adsorbed phase, where the *ϕ* angles are shown from −180 to 180° on the x‐axis, and *ψ* angles are shown in the same range on the y‐axis; (c) the per‐frame occupancy of the five most popular H‐bonds (donner‐acceptor) for C1 and C3‐l; (d) the propensity of MP‐11 residues to act as anchors in the simulation trajectories leading to C1 and C3‐l.

Armed with the above understanding, comment can be made on potential ways of enhancing the propensity of the C1 configuration at the expense of that of the C3‐l sub‐cluster. One is to facilitate an irreversible conformational reaction of C3‐l → C1. Unfortunately, this is very difficult to achieve as flipping from a C‐term down orientation to an N‐term down orientation faces very large steric barriers once peptide lockdown has been initiated. The only way of promoting C1 over C3‐l is, therefore, to widen the funnel to the former relative to that of the latter. This could potentially be achieved in a number of ways as outlined below.

The most obvious way to more realistically boost the propensity of the C1 cluster at the expense of the C3‐l sub‐cluster is to enhance the likelihood of the N‐term approaching the graphene first. As the anchoring data shown in Figure 7d shows, this could potentially be achieved by replacing the Val^11^ residue with another whose side chain is more polar whilst also changing the Glu^21^ residue for one that is nonpolar. This could be achieved by literally changing the residues through protein engineering, which is well practiced in the field. A simpler alternative, however, would be to reduce the pH to increase the fraction of microenzymes with a charged N‐term and uncharged C‐term. This may, however, also affect the charged status of the two propionate groups at the edge of the heme ring (and other residues), which may in turn affect the electron distribution across the heme group and, thus, its electronic coupling with the graphene and electron transfer rate. First‐principles calculations of the representative C1 conformation with protonated (i.e., uncharged) propionate groups does, indeed, suggest that the coupling is weakened whilst the equilibrium electron transfer rate is reduced by around half due to the larger energy gap between MP‐11 LUMO and graphene HOMO resulting from the change in heme charge state. This suggests that whilst making the solution more acidic is a simple way of increasing the propensity of the C1 configuration at the expense of the C3 configuration, gains made through this would be significantly offset by a lower electron transfer rate.

## 4 Conclusions

A new multiscale computational approach for bioelectrocatalytic systems has been described and used to study the MP‐11/saline/graphene system. The multiscale approach combines molecular dynamics (MD) simulation to elucidate in detail the adsorption of the heme‐containing MP‐11 microenzyme from a bulk saline solution on to a graphene/saline interface and the multiple resultant adsorbed states, and first principles methods—in particular density functional theory (DFT) and projector operator diabatization (POD) combined with the Markus‐Hush theory—to elucidate the electron transfer between the adsorbed MP‐11 and the graphene.

Consistent with prior studies by the authors, the MD simulations reveal a four‐stage adsorption mechanism initiated and guided by the electrostatic interactions between structured water layers at the graphene interface and specific parts of the MP‐11 molecule. These simulations also suggest that the heme ring can take on up to six different configurations in the space defined by the distance of the heme iron atom and the orientation of the normal to the heme ring and that of the graphene sheet (heme tilt angle).

Of the six heme configurations, the first principles calculations showed that two, accounting collectively for just under 13% of the heme configurational space (denoted C1 and C2), offer similarly good electron transfer rates due to both their proximity to the graphene (c6.5‐8 Å) *and* the heme ring being tilted relative to the graphene layer. A third configuration accounting for nearly 68% of the heme configurational space (denoted C3) presents, on the other hand, a far smaller electron transfer rate (around 25%) due to it sitting slightly further away from the graphene (c8.5‐9 Å) and, crucially, the heme ring being more edge‐on to the graphene sheet. The remaining three heme configurations, which see the heme ring at between 11 and 15 Å from the graphene, exhibit electron transfer rates that are 10^3–^10^4^ times smaller (i.e., negligible in any practical sense). Whilst there is no experimental data for the system considered here, the electron transfer rates evaluated here for the system and their variation with overpotential are comparable to experimental values and behavior for MP‐11 adsorbed on multi‐walled carbon nanotubes.

This report is concluded with an illustration of how the new multiscale approach may be used to optimize for electron transfer rate by focusing on what changes in the system may potentially enhance the propensity of the C1 configuration at the expense of the C3 configuration, which the first principles calculations suggest could potentially yield a 2–3 fold increase in electron transfer rate. The MD results suggest that this could be achieved by making the N‐term and C‐term more and less polar, respectively. This could be achieved by either protein engineering or, more simply, reducing the pH into the acidic range. However, first‐principles calculations suggests that the latter strategy may lead to a reduction in electron transfer rate, offsetting some (not all) of the gain obtained by increasing the propensity of the C1 configuration at the expense of the C3 configuration. Future reports will focus more on exploring such optimizations in detail.

While demonstrated here for MP‐11 on graphene, the framework is general and can be applied to other redox microenzymes, conductive electrode materials, and immobilization conditions. By building a quantitative bridge from the molecular characteristics to its device‐relevant electron‐transfer performance, and by translating that link into design strategies, it offers a rational alternative to the empirical development processes that have long dominated bioelectrocatalysis, biosensing and bioenergy. The methodology also offers a route in due course to the design of technologies based on large enzymes, which will be made possible by adoption of multiscale QM/MM schemes and machine‐learning predictions of electronic couplings [78]. Looking further ahead, exploration of the design‐space illustrated in Section 3.6 may be accelerated by machine learning approaches as proposed in Biggs and Mijajlovic [79], and realized more recently in, for example, the work of Njirjak et al. [80].

## Author Contributions

**Milan Mijajlovic**: Methodology, Software, Data curation, Investigation, Writing original draft, review and editing, Conceptualization, Visualization, Validation, Formal analysis and Supervision. **Cheng Hu**: Conceptualization, Methodology, Software, Data curation, Investigation, Validation, Formal analysis, Writing original draft, review and editing, Visualization, Funding acquisition, resources. **Yuhui Sun**: Investigation. **Matthew J. Penna**: Conceptualization, Investigation, Supervision. **Meisam V. Kiamahalleh**: Investigation. **Wenrong Yang**: Conceptualization, Resources, Writing review & editing. **Mark J. Biggs**: Conceptualization, Data curation, Formal analysis, Funding acquisition, Methodology, Project administration, Resources, Supervision, Writing original draft, review and editing.

## Conflicts of Interest

The authors declare no conflicts of interest

## Supporting information




**Supporting File**: advs76485‐sup‐0001‐SuppMat.docx.

**Supporting File**: advs76485‐sup‐0002‐VideoS1.mp4.

## Data Availability

The data that support the findings of this study are available from the corresponding author upon reasonable request. The CP2K input files, POD outputs, and the scripts used to extract the electronic couplings and compute the rate constants are openly available in a repository (https://github.com/Cheng75913/MP11‐Graphene_Charge_Transfer).
