# Supplementary material for: Multiscale Modelling of the Electrocatalysis of Microenzymes: Insights Into the Performance of Microperoxidase at an Aqueous/Graphene Interface and Their Exploitation to Improve Performance
Source: Adv Sci (Weinh). 2026 Jul 20:e76485. Online ahead of print. doi: 10.1002/advs.76485 (PMC13383154; doi:10.1002/advs.76485)
Supplement: Supplementary file 1 — Supporting File: advs76485‐sup‐0001‐SuppMat.docx. [file ADVS-9999-e76485-s002.docx]

Supporting Information

**Multiscale modelling of the electrocatalysis of microenzymes: Insights into the performance of microperoxidase at an aqueous/graphene interface and their exploitation to improve performance**

Milan Mijajlovic, Cheng Hu, Yuhui Sun, Matthew J Penna, Meisam V Kiamahalleh, Wenrong Yang, and Mark J Biggs*

**Supporting Figures**


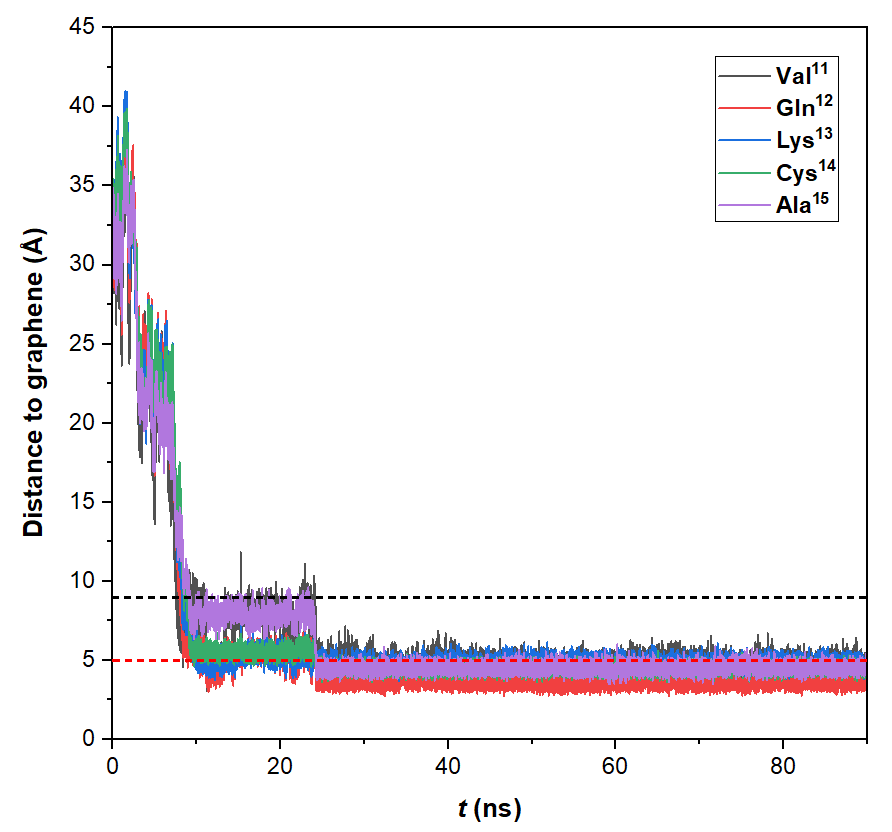


**Figure S1.** C^α^-to-graphene distance *vs*. time for the contacting residues of an exemplar trajectory, with horizontal reference lines at 5.0 and 7.7 Å indicating the first and second water layers.

**
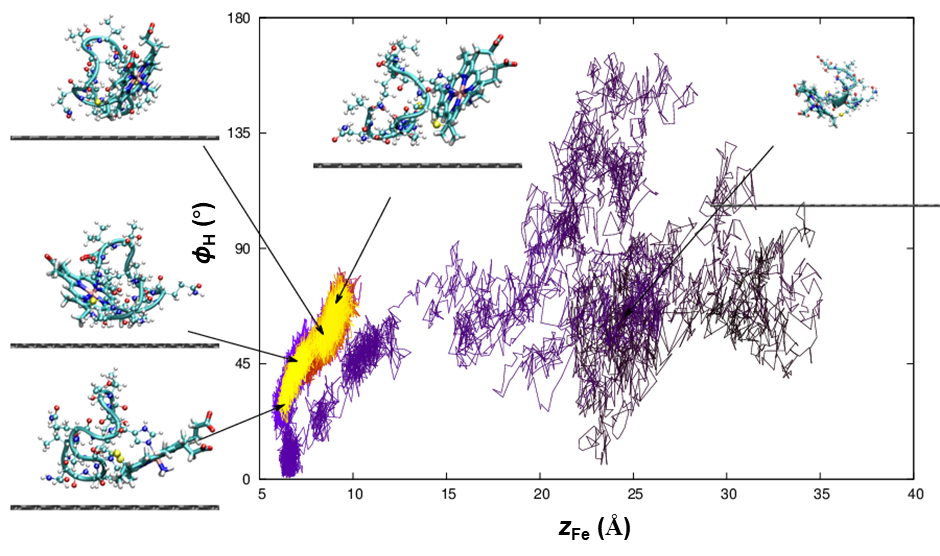
**

**Figure S2.** A trajectory of an exemplar simulation shown in the space of the two degrees of freedom used to describe the position and orientation of the heme group of the MP-11 molecule relative to the graphene layer, *z_Fe_* and *ϕ_H_*, respectively, as defined in Figure 7. The trajectory’s arrow of time is indicated by the variation in the line colour from black at the start to violet to orange to red and, finally, yellow. Snapshots of the MP-11 molecule along the trajectory are inset.


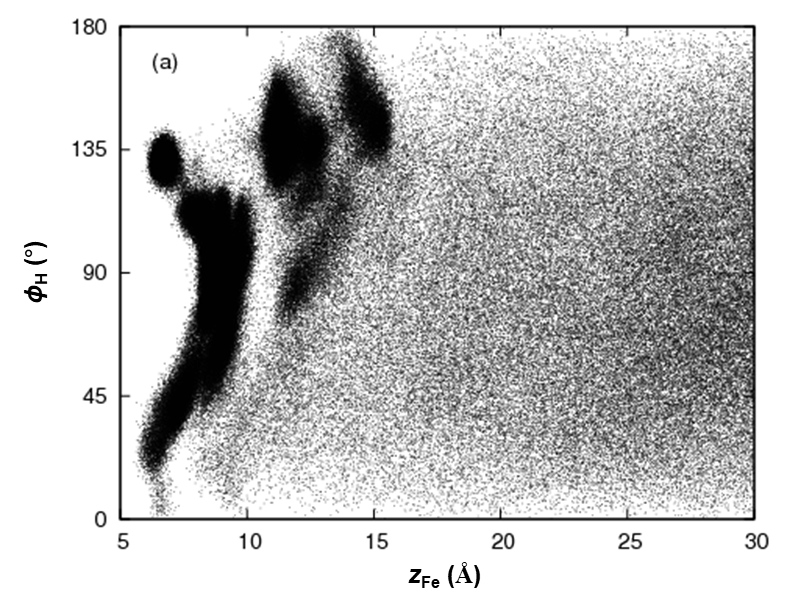


**Figure S3.** The recorded snapshots from all 57 simulations trajectories plotted in the *z_Fe_-ϕ_H_* space.


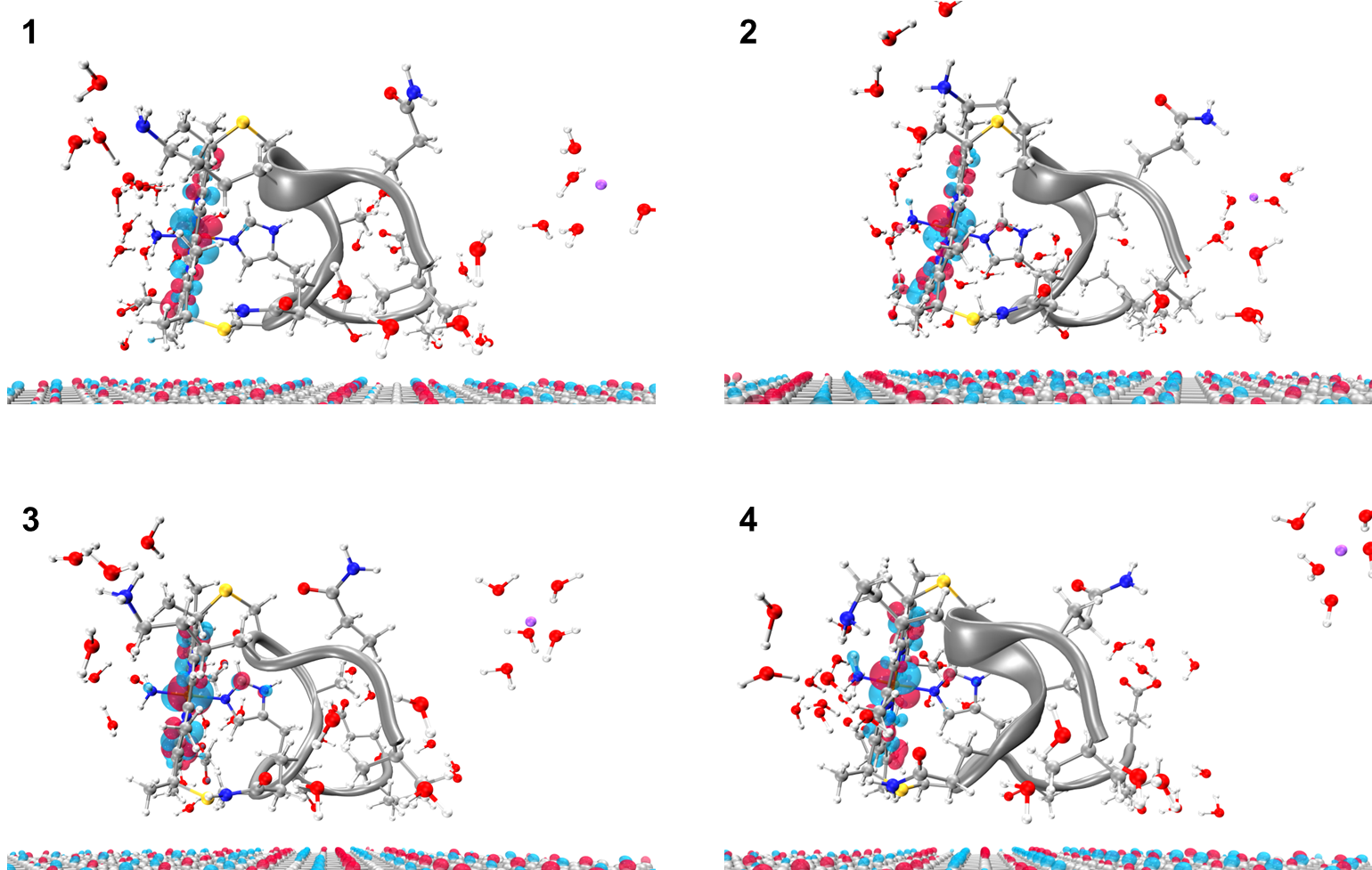


**Figure S4.** Structures taken from the four corners of the C3-l sub-cluster as marked in the probability density of heme configurations in the *z_Fe_-ϕ_H_* space shown in Figure 4b. The lowest unoccupied molecular orbital (LUMO) of MP-11 and the highest occupied molecular orbital (HOMO) of graphene, obtained from the POD analysis of electron transfer, are plotted for an isosurface value of 0.03.


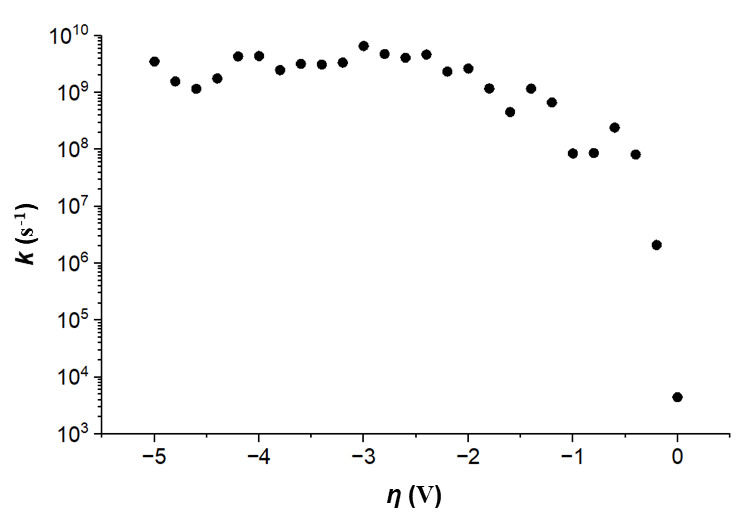


**Figure S5.** Calculated electron transfer rate constant (*k*) as a function of overpotential (*η*) for a representative conformation of heme configuration cluster C1.


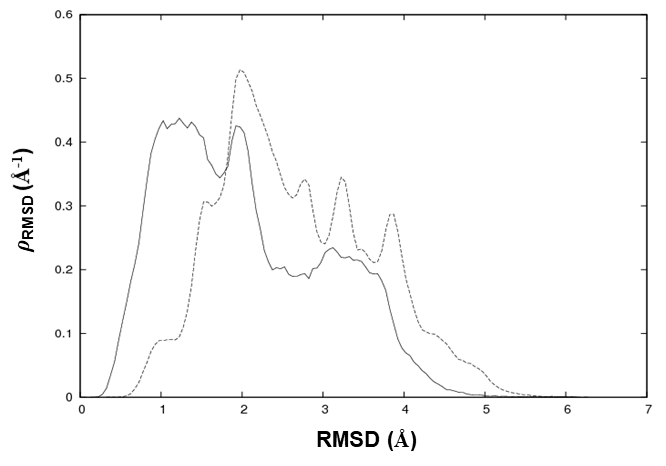


**Figure S6.** Probability density of finding a structure with a particular RMSD in the bulk (solid line) and the adsorbed phase (dashed line). For each simulation, the RMSD of its recorded snapshots is calculated using the starting structure as a reference.


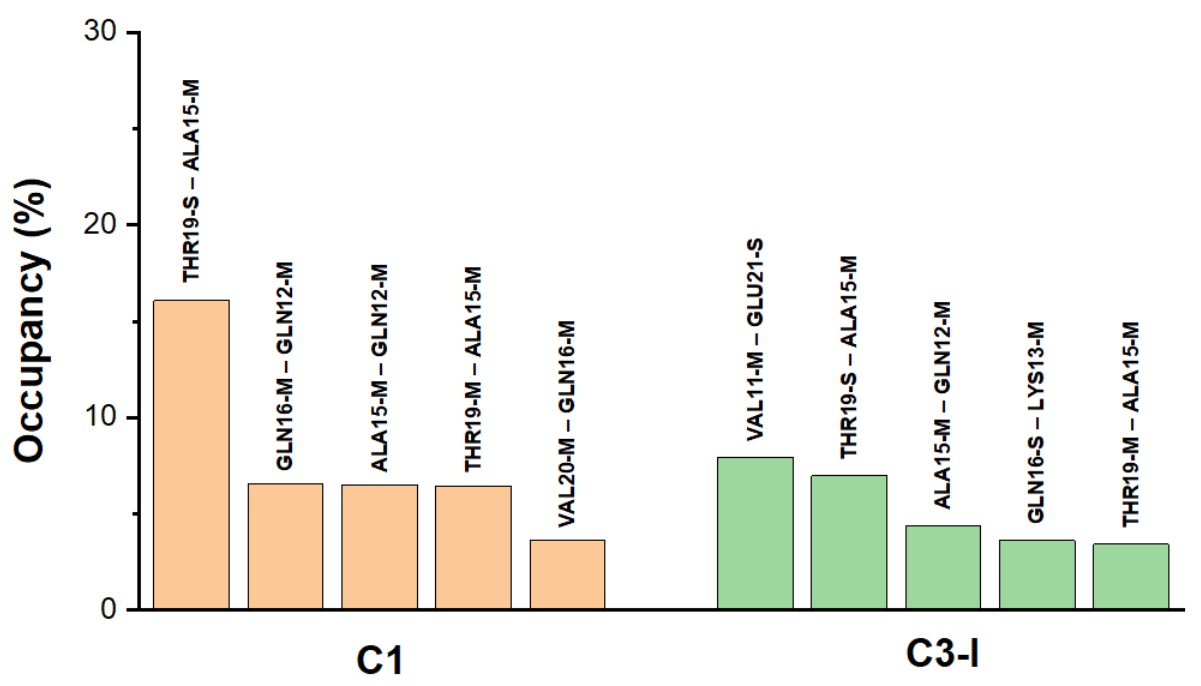


**Figure S7**. The per-frame occupancy of the five most popular H-bonds during the adsorption process of MP-11 from bulk to anchoring calculated from the simulation trajectories leading to C1 and C3-l respectively.

**Supporting Tables**

**Table S1** The variations of |*H*_ab_| and *k* within the sub-cluster of C3-l.

| **Structure** | ***z*_Fe_ (Å)** | ***ϕ* (°)** | $\bar{\left\vert\boldsymbol{H}_{\boldsymbol{ab}} \right\vert}$ **(eV)^*^** | ***k* (s^-1^, λ=0.3 eV)** | |
| --- | --- | --- | --- | --- | --- |
|  |  |  |  | *k*_0_ | *k*_max_ (×10^4^) |
| **0^**^** | 8.426 | 98.5 | 0.192 | 1150 | 3900 |
| **1** | 8.332 | 94.5 | 0.216 | 1660 | 4790 |
| **2** | 8.330 | 102.5 | 0.292 | 2290 | 5370 |
| **3** | 8.961 | 93.7 | 0.137 | 420 | 1030 |
| **4** | 9.037 | 99.2 | 0.160 | 977 | 1770 |

^*^Average |*H*_ab_| between MP-11 LUMO (located on the heme group) and the highest 10 occupied orbitals of graphene.

^**^Structure taken to represent the sub-cluster.
